# 1690-Fold enhanced electrochemiluminescence of gold nanoclusters *via* Zn^2+^ induced aggregation

**DOI:** 10.1039/d5sc06929g

**Published:** 2025-10-24

**Authors:** Yujiao Wang, Xuwen Gao, Qinqing Zhang, Xiaoxuan Ren, Guizheng Zou

**Affiliations:** a School of Chemistry and Chemical Engineering, Shandong University Jinan 250100 China zouguizheng@sdu.edu.cn +86-531-88361326

## Abstract

Herein, a zinc ion (Zn^2+^)-induced aggregation strategy is proposed for 1690-fold enhanced electrochemiluminescence (ECL) from d-penicillamine (DPA)-capped gold nanoclusters (AuNCs), and the aggregation-induced ECL (AIECL) is a multiplicative effect of two enhancing factors. One is the improved ligand rigidification of Zn^2+^-induced aggregation of AuNCs, *i.e.* Zn^2+^-AuNCs. The Zn^2+^ of Zn^2+^-AuNCs can reduce the vibration and rotation of DPA, suppress non-radiative decay, and result in ∼17-fold enhanced emission with photoluminescence (PL) as control. The other is the promoted electron transfer by Zn^2+^–DPA bonds, which can enhance charge annihilation with the assistance of energy level alignment between AuNCs and the Zn^2+^–DPA bond, further amplifying ECL by another ∼100-fold. The dual-enhanced AIECL strategy can be utilized to design even brighter ECL luminophores.

## Abstract 2

Schematic illustration of 1690-fold enhanced electrochemiluminescence of gold nanoclusters *via* Zn^2+^ induced aggregation.

## Graphical abstract

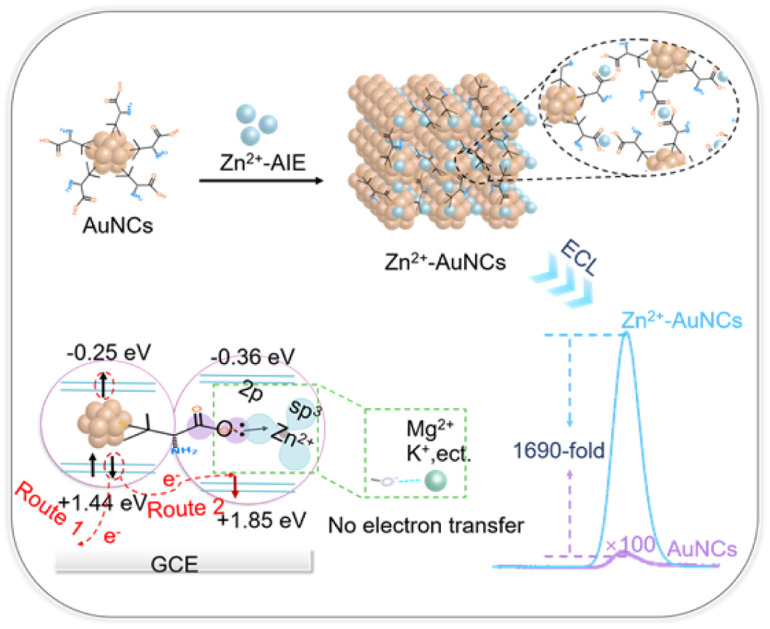

## Introduction

Electrochemiluminescence (ECL) is a powerful analytical technique with high sensitivity, low background noise, and rapid response,^1–3^ making it widely applicable in biomedical diagnostics,^4^ environmental monitoring,^5^ and food safety.^6^ ECL relies on the emission from electrochemically excited luminophores, offering distinct advantages over traditional fluorescence methods, such as minimal interference from scattered light and being free of external light sources.

The profound application of ECL is frequently postponed by its limited photons per luminophore, *i.e.* brightness, particularly the luminophore of noble metal nanoclusters (NCs), which normally suffer from severe unfavourable effects from non-radiative decay caused by ligand vibration and rotation.^7–9^

Recently, aggregation-induced emission (AIE) has attracted significant attention due to its ability to effectively enhance photoluminescence (PL) by restricting intramolecular motion.^10–12^ The aggregation of luminophores can reduce free rotation and vibration, suppressing non-radiative decay for enhanced PL.^8,13^ Aggregation-induced ECL (AIECL) might provide an alternative to the normal signal amplifying ECL strategy in ECL.^14–17^

Au nanoclusters (NCs) are biocompatible luminophores for bioassay.^18,19^ AuNCs can exhibit molecule-like behaviour, discrete electronic transitions and size-dependent luminescence.^20^ The PL of AuNC luminophores critically depends on the endogenous charge transfer behaviour under exogenous exciting conditions,^9,21^ including lifetimes,^22^ energy dissipation pathways (*e.g.*, radiative *vs.* non-radiative decay),^23^ and interactions.^24^ Although many metal ions can enhance the ECL of Au NCs *via* the AIE mechanism,^25,26^ the detailed mechanisms are still far from clear.

Among various metal ions, Zn^2+^ exhibits significant advantages due to its unique 3d^10^ electronic configuration and flexible coordination geometry. Zn^2+^ can form strong interactions with electron-rich carboxylate ligands of capping agents,^25^ including d-penicillamine (DPA). Bain *et al.* found that Zn^2+^-induced aggregation of AuNCs can result in 50-fold enhanced PL.^27^ To the best of our knowledge, the threshold fold value for AIE enhanced ECL is around 1200 (Table S1),^23,25,26,28–35^ while the threshold fold value for metal ion induced AIECL is only around 50.^26^ It is well known that the excited state of ECL is generated in an electrochemical electron transfer involved way, which is different from the photon induced electron transfer of PL.^8,36,37^ The electrochemical redox induced AIECL might be favorable for metal ions to modulate charge transfer pathways and achieve even higher ECL brightness. Inspired by these points, 1690-fold enhanced ECL from AuNCs to Zn^2+^-induced aggregates of AuNCs, *i.e.* Zn^2+^-AuNCs, is achieved by multiplying the effects of the improved ligand rigidification and the promoted electron transfer as well as charge annihilation within the aggregates in this case.

## Experimental

### Chemicals and materials

All chemical reagents were of analytical grade or higher. Hydrogen tetrachloroaurate(iii) trihydrate (HAuCl_4_·3H_2_O; 99.0%), d-penicillamine (DPA), borane-*tert*-butylamine complex (BTB; 95.0%), glycine (Gly; 99.0%) and 2-[4-(2-hydroxyethyl)piperazin-1-yl]ethanesulfonic acid (Hepes; 99.0%, 1 M) were purchased from Aladdin Industrial Co., Ltd (Shanghai, China). *p*-Aminobenzoic acid (ABA; 99.5%), zinc nitrate hexahydrate (Zn(NO_3_)_2_·6H_2_O; 99.0%), ethylenediaminetetraacetic acid (EDTA; 99.5%) and 2-dibutyaminoethanol (DBAE; 99.0%) were purchased from Sinopharm Chemical Reagent Co., Ltd (Shanghai, China). Potassium hexacyanoferrate (K_4_Fe(CN)_6_) and potassium ferricyanide (K_3_Fe(CN)_6_) were purchased from Xilong Chemical Co., Ltd (Shantou, China).

### Apparatus and measurements

PL spectra were recorded with an F-320 spectrofluorimeter (Tianjin Gangdong Sci. &Tech. Development Co., Ltd, China). PL lifetime and PL quantum yield (PLQY) were recorded on an FLS1000 fluorescence spectrometer (Edinburgh Instrument Co., Ltd, U.K.). UV-vis absorption was recorded on a TU-1901 spectrophotometer (Beijing Purkinje General Instrument Co., Ltd, China). The high-resolution transmission electron microscopy (HRTEM) images were taken on a JEM 2100 transmission electron microscope with an intermediate acceleration voltage of 300 kV (JEOL Co., Ltd, Japan). The matrix-assisted laser desorption ionization time of flight measured with a Bruker microflex LRF-MALDI TOF MS (Bruker Co., Ltd, Germany). The Fourier transform infrared spectroscopy (FT-IR) spectrum of powdered samples was recorded on an ALPHA II Fourier Transform Infrared research spectrometer (Bruker Co., Ltd, Germany). The energy dispersive spectrometer (EDS) pattern was recorded on an Oxford X-MaxTEM (Shanghai Oxford Instrument Technology Co., Ltd, China). X-ray photoelectron spectroscopy (XPS) was performed on a Thermo Scientific ESCALAB Xi+ using monochromatic Al Kα radiation (Thermo Fisher Scientific Co., Ltd, USA). Zeta potential (*ζ*) and dynamic light scattering (DLS) were measured on a NanoZS Zetasizer Nanoseries (Malvern Instruments Co., Ltd, Britain). Electron Paramagnetic Resonance (EPR) was recorded on a Bruker EMX PLUS spectrometer (Bruker Co., Ltd, Germany). Differential Pulse Voltammetry (DPV) and Cyclic Voltammetry (CV) were conducted with a CHI 1040C electrochemical analyzer (Shanghai Chenhua Instrument Co., Ltd, China). Electrochemical Impedance Spectroscopy (EIS) was carried out on a VersaSTAT 3 electrochemical analyzer (Princeton Instrument Co., Ltd, USA). Electrochemiluminescence (ECL) measurements were conducted on an MPI-EII ECL analyzer (Xi'an Remex Analytical Instrument Co., Ltd, China) with a glassy carbon working electrode, an Ag/AgCl (saturated KCl) reference electrode and a Pt wire counter electrode. ECL spectra were recorded on a GCFG-B ECL spectrum analyzer (Shandong GuoChen Biotech Co., Ltd, China). Ultraviolet photoelectron spectroscopy (UPS) was performed using a Thermo Fisher Scientific K-Alpha system (Thermo Fisher Scientific Co., Ltd, USA).

### Preparation of AuNCs and Zn^2+^-AuNCs

AuNCs and Zn^2+^-induced aggregates of AuNCs, *i.e.* Zn^2+^-AuNCs, were prepared according to a reference with modifications. Typically, HAuCl_4_·3H_2_O (96 mM, 200 μL) and DPA (100 mM, 400 μL) were introduced into 5 mL deionized water (DDW) in sequence under vigorous stirring. Then, the borane-*tert*-butylamine complex (BTB) (100 mM, 300 μL) was added dropwise to the mixture and the color gradually turned brown. 30 min later, pH was adjusted to 8.0 with 0.1 M NaOH, and the solution was stirred for 30 min to obtain crude AuNCs. 0.17 g zinc nitrate hexahydrate (Zn(NO_3_)_2_·6H_2_O) was introduced into the crude and allowed to react for 30 min at room temperature under stirring to form Zn^2+^-AuNCs. AuNCs and Zn^2+^-AuNCs were purified three times with isopropyl alcohol *via* centrifugation, redispersed in 5 mL DDW, and stored at 4 °C.

## Results and discussion

### Optical enhancement of Zn^2+^-AuNC aggregates

**Fig. 1:**
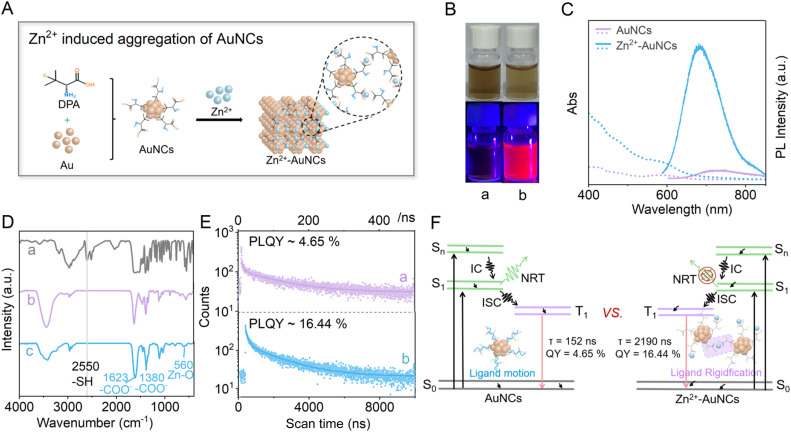
(A) Schematic illustration of Zn^2+^ induced aggregates of AuNCs; (B) photographs of (a) AuNCs and (b) Zn^2+^-AuNCs under ambient light (top) and UV irradiation (bottom); (C) UV-Vis absorption and PL spectra (*λ*_ex_ = 560 nm) of 1.6 mg mL^−1^ (a) AuNCs and (b) Zn^2+^-AuNCs in water; (D) FT-IR spectra of (a) DPA, (b) AuNCs and (c) Zn^2+^-AuNCs; (E) PL decay profiles of (a) AuNCs and (b) Zn^2+^-AuNCs; (F) scheme for the excited-state dynamics of AuNCs and Zn^2+^-AuNCs.

AuNCs and Zn^2+^-induced aggregates of AuNCs, *i.e.* Zn^2+^-AuNCs, are prepared according to a reference with modifications (Fig. 1A).^27^ The photophysical modulation of DPA-stabilized AuNCs upon Zn^2+^-induced aggregation is demonstrated in Fig. 1. Zn^2+^-AuNCs exhibit bright red emission centered at ∼680 nm (Fig. 1B, bottom right) under ultraviolet light irradiation. The absorption of AuNCs and Zn^2+^-AuNCs displays characteristic peaks at 465 nm and 560 nm, respectively. The absorption of Zn^2+^-AuNCs is significantly stronger than that of AuNCs, because the reduced interparticle distances within aggregates might enhance the local electric field.^38^ The PL of AuNCs (*λ*_ex_ = 560 nm) reaches a maximum at 730 nm with a full width at half maximum (FWHM) of 175 nm, while Zn^2+^-AuNCs exhibit 50 nm blue-shifted (*λ*_em_ = 680 nm) and 17-fold enhanced PL with a FWHM of 110 nm (Fig. 1C). The blue-shifted and narrowed PL originates from aggregation-induced changes in excited-state relaxation dynamics, which are typical features of AIE.^39^ The enhanced PL originates from the reduced motion of DPA ligands within the aggregation, which suppresses non-radiative decay.

According to Fig. 1D, the disappeared –SH vibration peak (2550 cm^−1^) of precursor DPA confirms that both AuNCs (curve b) and Zn^2+^-AuNCs (curve c) are stabilized by Au–S bonds.^40^ Zn^2+^-AuNCs exhibit some new peaks at 560 cm^−1^ (Zn–O vibration) and 1623 cm^−1^/1380 cm^−1^ (asymmetric/symmetric COO^−^ stretching), indicating the occurrence of coordination between Zn^2+^ and the carboxylate group of DPA. This coordination can form a rigid metal–ligand network that restricts the vibrational and rotational motions of capping agents,^41^ suppresses nonradiative decay pathways, and enhances the efficiency of radiative transition. Consequently, the PL of Zn^2+^-AuNCs is 17-fold higher than that of AuNCs.

### Morphological evolution, elemental composition and surface states of Zn^2+^-AuNCs

According to Fig. 1E, the triexponential decay profiles of AuNCs and Zn^2+^-AuNCs reflect multiple excited-state relaxation pathways. Zn^2+^-induced aggregation prolongs the fluorescence lifetime of AuNCs from 126.91 ns to 2083.04 ns (16.4-fold increase; Table S2). The dominant long-lived component in the Zn^2+^-AuNC decay curve indicates a more persistent excited state in aggregates, where ligand motions are effectively constrained, thereby extending the radiative lifetime. These results provide unambiguous evidence that interaction between Zn^2+^ and carboxylate ligands of DPA can restrict the motion of luminophores within aggregates (Fig. 1A & S1) and enhance the PL lifetime as well as excited-state efficiency (Fig. 1F).

**Fig. 2:**
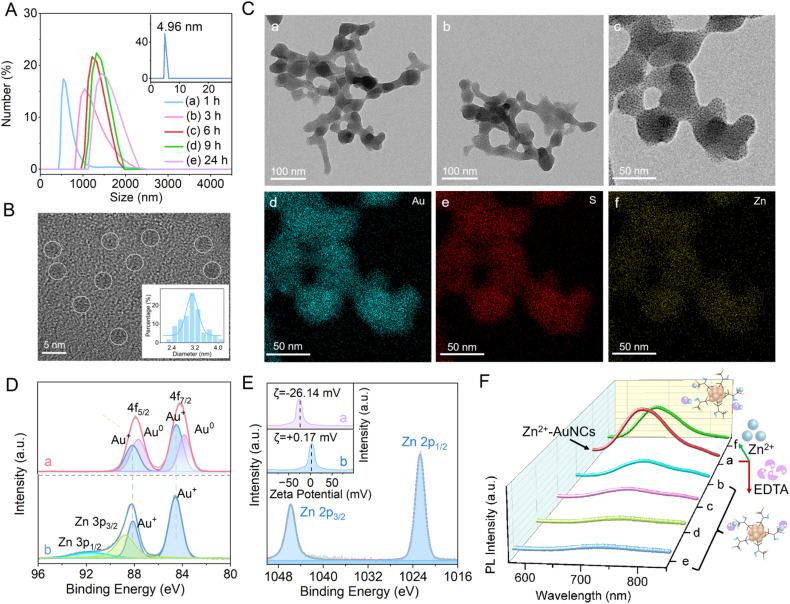
(A) Time-resolved DLS spectra of AuNCs after the addition of Zn^2+^; (B) HRTEM image of AuNCs; (C) HRTEM images of Zn^2+^-AuNCs and corresponding elemental mapping of Au, S and Zn in Zn^2+^-AuNCs; (D) XPS spectra for Au 4f of (a) AuNCs and (b) Zn^2+^-AuNCs; (E) XPS spectrum for Zn 2p of Zn^2+^-AuNCs; (F) effects of exogenous EDTA on PL of Zn^2+^-AuNCs *via* mixing EDTA and Zn^2+^-AuNCs at EDTA : Zn^2+^ (within Zn^2+^-AuNCs) molar ratios of (a) 0 : 1, (b) 0.25 : 1, (c) 0.50 : 1, (d) 0.75 : 1, and (e) 1 : 1 as well as (f) mixing EDTA, Zn^2+^-AuNCs and monodispersed Zn^2+^ at an EDTA : Zn^2+^ (within Zn^2+^-AuNCs) : Zn^2+^ (monodispersed) molar ratio of 1 : 1 : 1. Insets: (A) DLS spectrum of AuNCs; (B) size distribution pattern of AuNCs; (E) zeta potential profiles of (a) AuNCs and (b) Zn^2+^-AuNCs.

According to Fig. 2A, dynamic light scattering (DLS) patterns provide unambiguous evidence for the time-dependent size growth of Zn^2+^-AuNC aggregates. Upon adding Zn^2+^, the hydrated particle size of AuNCs experiences a rapid increase from 4.96 nm to ∼560 nm within 1 h (curve a). This growth process can continue over a long time, and the particle size can increase to ∼1400 nm at 24 h (curve e).

According to Fig. S2, both AuNCs and Zn^2+^-AuNCs exhibit clear XPS profiles of Au and S, while Zn^2+^-AuNCs demonstrate a clear XPS profile of Zn, indicating that Zn^2+^ is involved in the formation of Zn^2+^-AuNCs. The aggregation and evolution of AuNCs to Zn^2+^-AuNCs are further confirmed by HRTEM and elemental mapping characterization. According to Fig. 2B and C, AuNCs are spherical with an average diameter of 3.1 nm, Zn^2+^-AuNCs are net-like aggregates with Au, S and Zn elements uniformly distributed. The contents of Au, S and Zn elements within Zn^2+^-AuNCs are 48.4%, 34.8% and 16.8%, respectively (Fig. S3).

According to Fig. 2D, XPS profiles for Au 4f_7/2_ and 4f_5/2_ of AuNCs can be decomposed into two clear doublets: peaks at 84.5 as well as 88.4 eV for Au(i) and 83.9 as well as 87.9 eV for Au(0).^42^ XPS profiles for Au 4f_7/2_ and Au 4f_5/2_ of Zn^2+^-AuNCs merely exhibit one peak at 84.5 and 88.4 eV, respectively, indicating that only Au(i) remains in aggregates.

Zn^2+^-AuNCs not only exhibit distinct XPS profiles of Zn 3p_3/2_ at 88.8 eV and Zn 3p_1/2_ at 91.2 eV, indicating that the valence of Zn element within Zn^2+^-AuNCs is +2 (Fig. 2D), but also demonstrate clear XPS profiles of Zn 2p_3/2_ at 1021.5 and Zn 2p_1/2_ at 1043.7 eV (Fig. 2E), which are related to Zn 2p of Zn–O,^25,43^ indicating that electrostatic attraction plays an important role in the interaction and coordination between Zn^2+^ and the –COO^−^ group of DPA.^44^

The zeta potential of AuNCs is −26.14 mV, while that of Zn^2+^-AuNCs is +0.17 mV (inset of Fig. 2E), providing clear evidence that the coordination of Zn^2+^–COO^−^ within Zn^2+^-AuNCs can neutralize the surface charge of aggregates.^45^ To confirm the charge neutralization role of Zn^2+^ in Zn^2+^-AuNCs, EDTA competition experiments are conducted, because EDTA is a common chelating agent for Zn^2+^.^46^ According to Fig. 2F, upon introducing EDTA into Zn^2+^-AuNCs, the PL of Zn^2+^-AuNCs decreased dramatically with an increased molar ratio of EDTA : Zn^2+^ from 0 : 1 to 1 : 1 (curves a–e). When the molar ratio of EDTA : Zn^2+^ is 1 : 1, only 5.9% PL remains. Interestingly, the decreased PL can be restored to 70.6% of its original value *via* further introducing exogenous Zn^2+^ into the mixture with a final molar ratio of EDTA : Zn^2+^ at 1 : 2 (curve f). The reversible PL confirms that Zn^2+^ is an aggregation trigger of Zn^2+^-AuNCs, while the aggregation trigger can be utilized to modulate excited-state efficiency and emission intensity.

### Electrochemistry and ECL of Zn^2+^-AuNCs

**Fig. 3:**
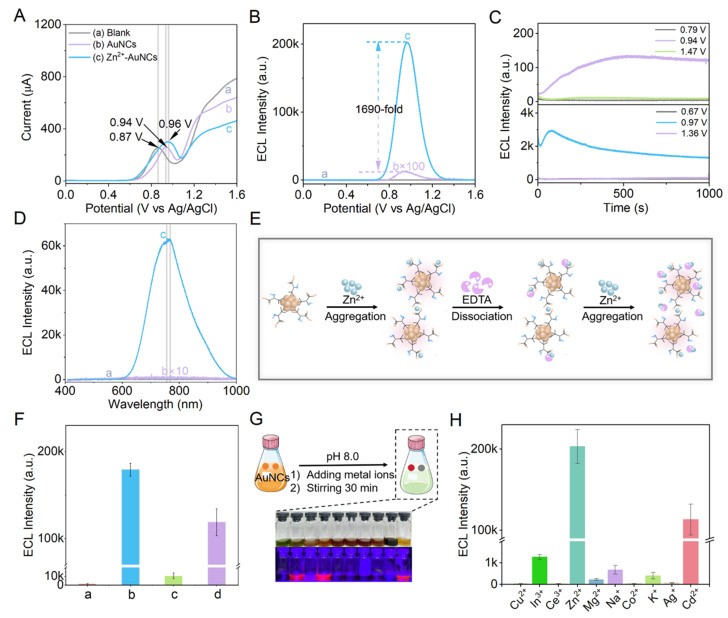
(A) DPV of the GCE in (a) 0.1 M pH 7.4 Hepes, (b) (a) + 1.6 mg per mL AuNCs, and (c) (a) + 1.6 mg per mL Zn^2+^-AuNCs; (B) ECL intensity–potential profiles of the GCE in (a) 0.1 M pH 7.4 Hepes containing 10 mM DBAE, (b) (a) + 1.6 mg per mL AuNCs, and (c) (a) + 1.6 mg per mL Zn^2+^-AuNCs; (C) ECL transient profiles of the GCE in 0.1 M pH 7.4 Hepes containing 10 mM DBAE and (a) 1.6 mg per mL AuNCs with the potential held at 0.79, 0.94, and 1.47 V, and (b) 1.6 mg per mL Zn^2+^-AuNCs with the potential held at 0.67, 0.97, and 1.36 V; (D) ECL spectra of the GCE in 0.1 M pH 7.4 Hepes containing (a) 10 mM DBAE, (b) (a) + 1.6 mg per mL AuNCs, and (c) (a) + 1.6 mg per mL Zn^2+^-AuNCs; (E) schematic of aggregation, disaggregation, and re-aggregation of 1.6 mg per mL AuNCs regulated with 100 mM Zn^2+^ and 100 mM EDTA; (F) the maximum ECL intensity of the GCE in 0.1 M pH 7.4 Hepes containing 10 mM DBAE and (a) 1.6 mg per mL AuNCs, (b) (a) + 100 mM Zn^2+^, (c) (b) + 100 mM EDTA, and (d) (c) + 100 mM Zn^2+^; (G) schematic of procedures for adding 100 mM of different metal ions to 1.6 mg per mL AuNCs and corresponding photographs under ambient light (top) and UV irradiation (bottom); (H) effects of metal ion precursors on the maximum ECL intensity of metal ion induced aggregates of 1.6 mg per mL AuNCs. Scan rate: 50 mV s^−1^.

Fig. 3A presents DPV profiles of AuNCs and Zn^2+^-AuNCs in 2-[4-(2-hydroxyethyl)piperazin-1-yl]ethanesulfonic acid (Hepes). Hepes exhibits a broad oxidative peak around 0.87 V (*vs.* Ag/AgCl) upon electrochemical oxidation beyond ∼0.6 V. Notably, the incorporation of AuNCs into Hepes induces a distinct oxidative peak at 0.94 V, clearly separated from the intrinsic Hepes peak at 0.87 V. Likewise, the presence of Zn^2+^-AuNCs in Hepes generates another distinct peak at 0.96 V, which is further distinguishable from the background Hepes oxidation. These shifted and well-defined peaks unambiguously confirm the electrochemical oxidation of AuNCs and Zn^2+^-AuNCs, rather than being dominated by Hepes-related oxidation processes.

AuNCs and Zn^2+^-AuNCs can be electrochemically oxidized at a similar potential. The oxidative current of Zn^2+^-AuNCs is much stronger than that of AuNCs, indicating that coordination of Zn^2+^–COO^−^ within Zn^2+^-AuNCs is favorable for hole injection. The enhanced hole injection can be anticipated to enhance the AIE of Zn^2+^-AuNCs in an electrochemical oxidation excited way.

Fig. 3B presents the co-reactant ECL profiles of AuNCs and Zn^2+^-AuNCs in Hepes in the presence of the co-reactant 2-dibutyaminoethanol (DBAE). Zn^2+^-AuNCs exhibit ECL from 0.67 to 1.36 V with a maximum emission potential of around 0.97 V, which is consistent with the oxidative potential of Zn^2+^-AuNCs. AuNCs exhibit ECL from 0.79 to 1.47 V with a maximum emission potential of around 0.94 V, which is also consistent with the oxidative potential of AuNCs. All these data prove that both AuNCs and Zn^2+^-AuNCs can be electrochemically injected with holes at the anode and then couple the electrons of reducing radicals for ECL, in which the reducing radicals are generated *via* oxidation of DBAE.^47^

The ECL of Zn^2+^-AuNCs is 1690-fold higher than that of AuNCs (Fig. 3B), and the enhanced factor of ECL from AuNCs to Zn^2+^-AuNCs is about 100-fold higher than that of the enhanced factor (∼17-fold) of PL (Fig. 1C). All these data prove that the electrochemical oxidation involving excitation is more favorable for enhancing the AIE of Zn^2+^-AuNCs than the normal photon excitation. The charge injection, transfer and annihilation within Zn^2+^-AuNCs are more efficient and favorable for the generation of emission than AuNCs. Additionally, after 10 consecutive cyclic potential scans on Zn^2+^-AuNCs, the maximum ECL intensity gradually decreases with an increase in the number of cycles (Fig. S4). This phenomenon may be attributed to the slight dissociation of some coordination bonds between Zn^2+^ and the carboxylate group of DPA under repeated potential scanning. This structural change leads to partial dispersion of the aggregated nanoclusters, which in turn weakens the charge transfer efficiency between the clusters. Notably, the ECL intensity of Zn^2+^-AuNCs still remains more than 100 times higher than the initial ECL intensity of AuNCs, fully demonstrating the significant core advantage of Zn^2+^-AuNCs in terms of ECL performance.

According to Fig. 3C, ECL is immediately generated by holding Zn^2+^-AuNCs at 0.97 V (curve c) and AuNCs at 0.94 V (curve b) respectively, and the ECL responses of AuNCs and Zn^2+^-AuNCs are rapid. In the case of holding Zn^2+^-AuNCs at 0.67 V and 1.36 V, or holding AuNCs at 0.79 V and 1.47 V, no obvious ECL is observed, and the ECL of AuNCs and Zn^2+^-AuNCs is highly potential-selective.

According to Fig. 3D, the ECL spectrum of Zn^2+^-AuNCs/DBAE exhibits a peak of 761 nm with a FWHM of 161 nm, and the ECL spectrum of AuNCs/DBAE exhibits a peak of 765 nm with a FWHM of 362 nm. The improved ECL monochromaticity from AuNCs to Zn^2+^-AuNCs indicates that some surface defects are removed *via* Zn^2+^-induced aggregation.^48^

### Enhanced mechanism for the AIECL of Zn^2+^-AuNCs

Compared to AuNCs, Zn^2+^-AuNCs exhibit 1690-fold enhanced ECL and 17-fold enhanced PL (Fig. 1C and 3B). The AIE of ECL, *i.e.* excitation induced by electrochemical oxidation, can result in about a 100-fold further enhancement factor of AIE compared to that of PL. The 100-fold further enhanced factor might be related to oxidation involved charge injection as well as the charge transfer and annihilation within Zn^2+^-AuNCs, and the reason lies in two aspects: one is that PL is the emission generated *via* photoexcitation, and its enhancement is mainly governed by the removed non-radiative decay of excited states;^36^ the other is that ECL is the emission generated *via* redox related charge injection, transfer and annihilation. Its enhancement is governed by both the removed non-radiative decay of excited states and the efficiency of charge injection, transfer, and annihilation.^49^

The EDTA competition experiment demonstrates reversible dissociation/aggregation of Zn^2+^-AuNCs: adding EDTA triggers dissociation by chelating Zn^2+^, while re-adding Zn^2+^ allows rebinding to AuNCs, causing reaggregation (Fig. 3E). According to Fig. 3F, the co-reactant ECL of AuNCs/DBAE in Hepes buffer is very weak (column a). Upon introducing Zn^2+^ into the same Hepes buffer containing both AuNCs and DBAE, greatly enhanced AIECL of Zn^2+^-AuNCs/DBAE is conveniently achieved (column b), indicating that Zn^2+^-induced aggregation of AuNCs is robust and can be extensively utilized to achieve AIECL under varied conditions. Although Zn^2+^-AuNCs can be efficiently achieved *via* mixing AuNCs and Zn^2+^ in Hepes in the presence of DBAE, further introducing DBAE into the solution would quench the AIECL by 93.8% (column c), because EDTA can disrupt the coordination bond of Zn^2+^ and –COO^−^. The integrity of Zn^2+^–COO^−^ is essential for efficient AIECL.

Upon further introducing Zn^2+^ into the above mixture, the subsequent Zn^2+^ replenishment can restore the AIECL to 61.6% of its original maximum (column d), and the AIECL of Zn^2+^-AuNCs is also chemically reversible, similar to corresponding PL (Fig. 2F). As demonstrated in Fig. 1A and Table S2, the interaction between Zn^2+^ and carboxylate ligands of DPA can restrict the motion of luminophores within aggregates, prolong PL lifetime by ∼16.4-fold, and result in ∼17-fold enhanced PL for Zn^2+^-AuNCs. It is clear that the motion of luminophores within aggregates might also only enhance the AIECL of Zn^2+^-AuNCs by the limited ∼17-fold, in a way similar to PL. Anyhow, the coordination of the Zn^2+^–COO^−^ structure is crucial to achieve efficient AIECL from Zn^2+^-AuNCs.

Upon the addition of different metal ion precursors to the AuNCs, the respective systems exhibit distinct characteristic fluorescence emissions under UV light (Fig. 3G). Fig. 3H further illustrates the impact of these metal ion precursors on the AIECL of AuNC related aggregates. Because only the 3d^10^ electronic configuration can form stable tetrahedral/octahedral coordination with –COO^−^*via* a spherical electron cloud (free of Jahn–Teller distortion), which is favorable for enhancing ECL *via* both ligand rigidification and electron transfer optimization, the metal ion induced AIECL in this case is highly selective to Zn^2+^. The Zn^2+^ (3d^10^) precursor enhances the AIECL of aggregates by 1690-fold, and the Cd^2+^ (4d^10^) precursor enhances the AIECL of aggregates by 878-fold due to the similar electronic structure to Zn^2+^ (3d^10^), and the obviously lowered enhancement performance was due to its larger ionic radius and weaker Lewis acidity. All the other metal ions of precursors fail to induce obvious AIECL, because Cu^2+^ (3d^9^) suffers from Jahn–Teller-distorted coordination and energy dissipation,^50^ Co^2+^ (3d^7^) in a high-spin state promotes non-radiative transitions *via* spin–orbit coupling,^51^ main-group metal ions (Mg^2+^, Na^+^, and K^+^) normally form weak electrostatic interactions, and Ag^+^ (4d^10^) exhibits poor binding ability with hard bases (–COO^−^) as a soft acid (Hard–Soft Acid–Base theory).

**Scheme 1:**
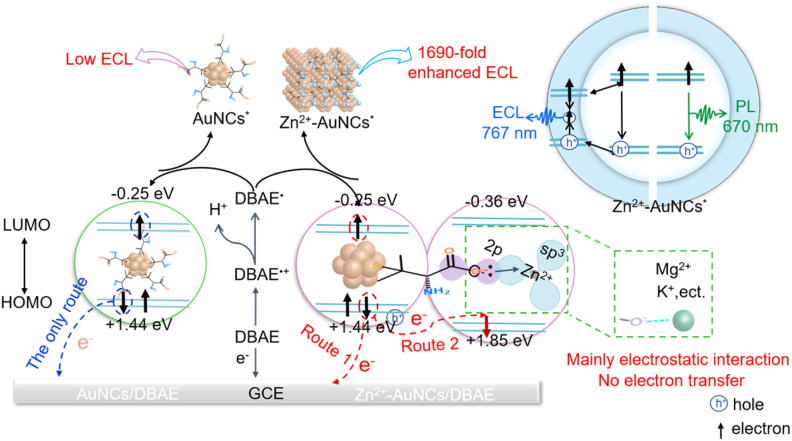
Mechanistic illustration for multiplicative and dual-enhanced AIECL of Zn^2+^-AuNCs.

As shown in Scheme 1, 1690-fold enhanced AIECL from AuNCs to Zn^2+^-AuNCs is a kind of multiplicative effect of these two factors: one is the ∼17-fold enhancement attributed to ligand rigidification *via* the coordination of Zn^2+^ and –COO^−^; the other is the ∼100-fold enhancement attributed to the promoted efficiency of electron injection, transfer, and annihilation *via* the coordination of Zn^2+^ and –COO^−^. The multiplicative effects of the two factors (17 × 100) ultimately achieve ∼1690-fold enhanced AIECL. It should be noted that multiplicative is used here to describe the combined contribution of rigidification and charge-transfer effects, not to imply their strict independence; these two effects are likely correlated, as structural rigidification may alter the local electronic environment to affect charge-transfer processes, and efficient charge transfer may in turn influence the stability of the rigidified structure.

### The formation of coordination bonds in Zn^2+^-AuNCs

Beyond structural evidence, the electron transfer thermodynamics within Zn^2+^–DPA bonds and its role in the enhanced AIECL are further explored with the Rehm–Weller equation (eqn (1)).^52^

1 Δ*G* = *E*^(donor)^_ox_ − *E*^(acceptor)^_red_ − *E*_exit_ + Δ*E*_sol_

where *E*^(acceptor)^_red_ and *E*^(donor)^_ox_ correspond to the reduction potential of Zn^2+^ (−0.50 V, curve a, Fig. S5) and the oxidation potential of AuNCs (0.92 V, curve b, Fig. S5), respectively. *E*_exit_ represents the electronic excitation energy of AuNCs (1.99 eV, Fig. S6), which is determined from the intersection (*λ*_is_) of the photoexcitation and photoluminescence spectra of AuNCs (*E*_excit_ = 1240/*λ*_is_). Δ*E*_sol_ represents the solvent-associated coulombic binding energy, which is considered negligible in aqueous solution. The calculated Δ*G* of −1.57 eV indicates thermodynamically favorable electron transfer from AuNCs to Zn^2+^–DPA. It should be noted that although the Rehm–Weller equation enables effective quantification of the thermodynamic feasibility of charge-transfer processes in this work, it represents an approximation when applied to Zn^2+^-AuNCs. This approximation arises primarily from the surface heterogeneity of the nanoclusters, a characteristic that can lead to minor discrepancies between the calculated and actual charge-transfer free energies.

The energy level alignment between AuNCs (1.44 eV/−0.25 eV, Fig. S7A) and Zn^2+^–DPA (1.85 eV/−0.36 eV, Fig. S7B) facilitates efficient electron transfer (detailed calculations are provided in the SI).^52^ Under anodic polarization, electrochemical oxidation of AuNCs promotes electron transition from the HOMO to the LUMO, forming an excited state. Due to the significant disparity in the HOMO energy levels between AuNCs (1.44 eV) and Zn^2+^–DPA (1.85 eV), with an energy difference of 0.41 eV, electrons spontaneously transfer from the HOMO of AuNCs to that of Zn^2+^–DPA. This process generates holes in the HOMO of AuNCs, thereby enhancing the efficiency of hole injection from the electrode into AuNCs (eqn (3)). Concurrently, the transfer of electrons to Zn^2+^–DPA reduces non-radiative electron–hole recombination within AuNCs, promoting the formation of excited states (Zn^2+^-AuNCs*) through electrochemical reactions (*e.g.*, the oxidative–reductive annihilation between DBAE˙ and AuNCs^+^, eqn (4)). Ultimately, the radiative transition of electrons from the LUMO to the HOMO (eqn (5)) is enhanced, leading to a substantial improvement in ECL efficiency.

Mott–Schottky analysis (Fig. S8) confirms the n-type semiconductor behavior of Zn^2+^-AuNCs, as evidenced by the positive slope of the curve. This indicates that electrons are the majority charge carriers, and Zn^2+^-AuNCs function as electron donors during electrochemical oxidation. Specifically, under anodic polarization, electrons in the conduction band of n-type Zn^2+^-AuNCs are injected into the electrode, leaving behind holes in the HOMO. These holes facilitate charge separation and suppress non-radiative recombination with electrons. Concurrently, electron paramagnetic resonance (EPR) spectroscopy (*g* = 2.0029, Fig. S9) reveals sulfur vacancies, which act as electron traps to stabilize the electron-depleted state and further enhance the efficiency of hole-driven electrochemical reactions. As an electron donor, Zn^2+^-AuNCs readily lose electrons from their HOMO orbitals to generate holes, while sulfur-based surface defects capture electrons, suppress recombination, and prolong the lifetime of excited states. This synergistic effect stabilizes excited states and enhances radiative transition efficiency, thereby facilitating ECL enhancement.

The multiplicative and dual-enhanced AIECL mechanism of Zn^2+^-AuNCs is illustrated in Scheme 1, and the corresponding charge transfer route is presented as the following equations:

2 DBAE − e → DBAE˙ + products

3 Zn^2+^-AuNCs − e → Zn^2+^-AuNCs^+^

4 Zn^2+^-AuNCs^+^ + DBAE˙ → Zn^2+^-AuNCs*

5 Zn^2+^-AuNCs* → Zn^2+^-AuNCs + hv (761 nm)

Upon the application of a positive potential, DBAE can be electrochemically oxidized to generate the reducing radicals DBAE˙ (eqn (2)).^47^ Holes are injected into the HOMO of Zn^2+^-AuNCs, resulting in hole-injected Zn^2+^-AuNCs (Zn^2+^-AuNCs^+^) (eqn (3)). DBAE˙ could inject an electron onto Zn^2+^-AuNCs^+^, which forms the excited states of Zn^2+^-AuNCs (Zn^2+^-AuNCs*) (eqn (4)), and ultimately facilitate the efficiency of oxidative-reduction ECL (eqn (5)).

## Conclusions

Multiplicatively enhanced AIECL by 1690-fold with two different enhancement factors is extensively explored with Zn^2+^-induced aggregates of Zn^2+^-AuNCs as a model. The coordination between Zn^2+^ and –COO^−^ groups of DPA can form a kind of Zn^2+^–DPA bond, which not only reduces the vibration and rotation of luminophores and results in ∼17-fold enhanced AIE emission with PL as a control, but also can work as a bridge to promote electrochemical redox-involved electron injection, transfer, and charge annihilation within aggregates, further multiplicatively enhancing AIE emission of ECL by another ∼100 fold. The multiplicative and dual-enhanced AIECL of Zn^2+^-AuNCs is highly selective for the electronic configuration of metal ion precursors, which is promising for establishing a novel methodology for optimizing the luminescent performance of nanomaterials.

## Author contributions

Y. W. conceived the concept, designed the experiments and synthesized the materials. Y. W. and X. G. performed material, electrochemical, and ECL characterizations. Q. Z., X. R., and G. Z. assisted in the interpretation of results. Y. W. and G. Z. wrote the paper and supervised the work. All authors gave their approval to the final manuscript.

## Conflicts of interest

There are no conflicts to declare.

## Supplementary Material

SC-016-D5SC06929G-s001

## Data Availability

The author can confirm that the data supporting the findings of this study can be obtained within the article (or in its supplementary information (SI)). Supplementary information is available. See DOI: https://doi.org/10.1039/d5sc06929g.
