# Supplementary material for: 1690-Fold enhanced electrochemiluminescence of gold nanoclusters via Zn2+ induced aggregation
Source: Chem Sci. 2025 Oct 24;16(47):22723–31. doi: 10.1039/d5sc06929g (PMC12573880; doi:10.1039/d5sc06929g)
Supplement: SC-016-D5SC06929G-s001 [file SC-016-D5SC06929G-s001.pdf]

Supplementary Information (SI) for Chemical Science.
This journal is © The Royal Society of Chemistry 2025

Supporting Information for

1690-Fold Enhanced Electrochemiluminescence of Gold Nanoclusters via Zn^{2+} Induced Aggregation

Yujiao Wang[a], Xuwen Gao[a], Qinqing Zhang[a], Xiaoxuan Ren[a], Guizheng Zou*[a]

a Y. Wang, X. Gao, Q. Zhang, X. Ren, G. Zou*

School of Chemistry and Chemical Engineering

Shandong University

250100 Jinan, China

E-mail: zouguizheng@sdu.edu.cn

Contents

(1) The measure process of ECL potential-intensity profiles S2

(2) Comparative analysis of (aggregation-induced ECL) AIECL systems enhancement....S2

(3) PL lifetime parameters of AuNCs and Zn^{2+}-AuNCs....S3

(4) Schematic illustration of Zn^{2+}-induced aggregation of AuNCs....S3

(5) XPS spectra of AuNCs and Zn^{2+}-AuNCs....S3

(6) EDS pattern of Zn^{2+}-AuNCs....S4

(7) ECL stability of Zn^{2+}-AuNCs....S4

(8) Cyclic voltammogram of Zn^{2+}-DPA and AuNCs....S5

(9) PL excitation and emission spectra of AuNCs....S5

(10) Investigation of the band gap (E_g) and energy level....S5

(11) Mott-Schottky curve of Zn^{2+}-AuNCs....S6

(12) EPR spectrum of Zn^{2+}-AuNCs....S6

(13) Calculations of energy levels....S6

(1) The measure process of ECL potential-intensity profiles

The ECL potential-intensity profiles were measured using a CHI 1040C electrochemical workstation coupled with an MPI-EII ECL analyzer (Xi'an Remex Analytical Instruments Co., Ltd., China).

Briefly, a certain concentration of ECL nano-emitters was monodispersed in 4 mL of pH 7.4 Hepes buffer, followed by adding 10 mM coreactant DBAE. With electrode potential control, DBAE and the nano-emitters undergo electrochemical oxidation or reduction at specific potentials; DBAE generates reactive intermediates via these processes, which drive the nano-emitters to an excited state through electron transfer. When the excited-state nano-emitters return to the ground state, they emit electrochemiluminescence with varying intensities.

Due to differences in redox properties, distinct ECL intensities are observed for different nano-emitters at various potentials. The luminescence signals are detected, converted, and amplified by an EMCCD (electron-multiplying charge-coupled device), yielding the relationship between potential and ECL intensity, i.e., the ECL potential-intensity profiles.

(2) Comparative analysis of (aggregation-induced ECL) AIECL system Enhancement

Table S1. Comparative analysis of a AIECL system enhancement.

System	Enhancement Factor (fold)	Triggering Potential (V vs. Ag/AgCl)	Ref.
[Ir(tpy)(bbbi)]	39	+1.2	28
GSH-AgNCs-ZIF-8	2.5	--	29
Zn-TCPP/UiO-66-NH_2	31.9	-1.9	30
$[Ru(phen)_2(phen\text{-}O_2)](PF6)_2$	16	+1.3	31
TTA-TAPE	206	+1.0	32
AuATP NCs (Ca^{2+})	50	+1.4	26
AuNPs@MXene	7.5	-1.9	33
TPE NAs	9.6	-2.0	34
Zn-PTC	1.6	-1.55	35
Zn^{2+}-GCA	2.7	-2.0	25
ATT-AuNCs	1200	+1.17	23
Zn^{2+}-AuNCs	1690	+0.97	This work

(3) PL lifetime parameters of AuNCs and Zn^{2+}-AuNCs

Table S2. PL Lifetime parameters for AuNCs (λ_{em} = 730 nm) and Zn^{2+}-AuNCs (λ_{em} = 680 nm)

Sample	τ_1/ns	A_1/%	τ_2/ns	A_2/%	τ_3/ns	A_3/%	τ_{av}/ns	χ^2
AuNCs	0.35	0.65	16.25	58.24	144.54	41.11	126.91	0.91
Zn^{2+}-AuNCs	2086.91	98.23	258.13	1.59	126.82	0.18	2083.04	0.97

The PL decay kinetics of AuNCs and Zn^{2+}-AuNCs were analyzed using triexponential fitting. The decay profiles were described by the function $I(t) = \sum A_i \exp(-t/\tau_i)$, where τ_i represents the distinct lifetime components and A_i corresponds to their respective amplitudes.

(4) Schematic illustration of Zn^{2+}-induced aggregation of AuNCs

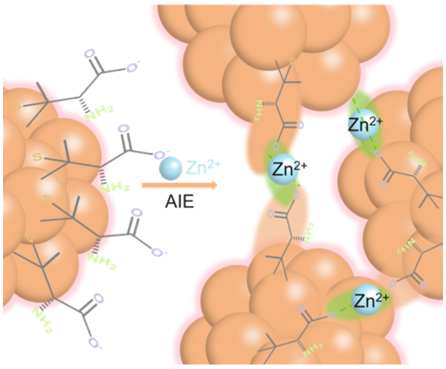

Figure S1. Schematic illustration of Zn^{2+}-induced aggregation of AuNCs. At pH 8.0, the carboxyl groups (-COO^-) on the surface of AuNCs re electrostatically attracted to Zn^{2+} cations, driving the spatial proximity of individual AuNCs. Subsequently, coordination bonds are established between the Zn^{2+} and -COO^-, enabling the aggregation of AuNCs through a coordination chemistry.

(5) XPS spectra of AuNCs and Zn^{2+}-AuNCs

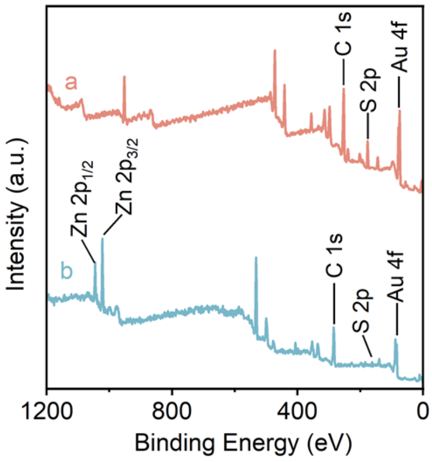

Figure S2. Survey XPS spectra of (a) AuNCs and (b) Zn^{2+}-AuNCs.

(6) EDS pattern of Zn^{2+}-AuNCs

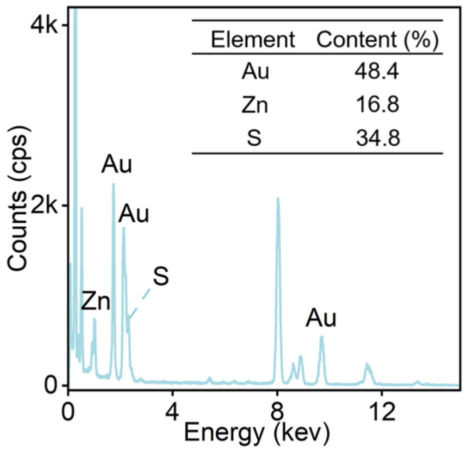

Figure S3. EDS pattern of Au, S and Zn in Zn^{2+}-AuNCs.

(7) ECL stability of Zn^{2+}-AuNCs

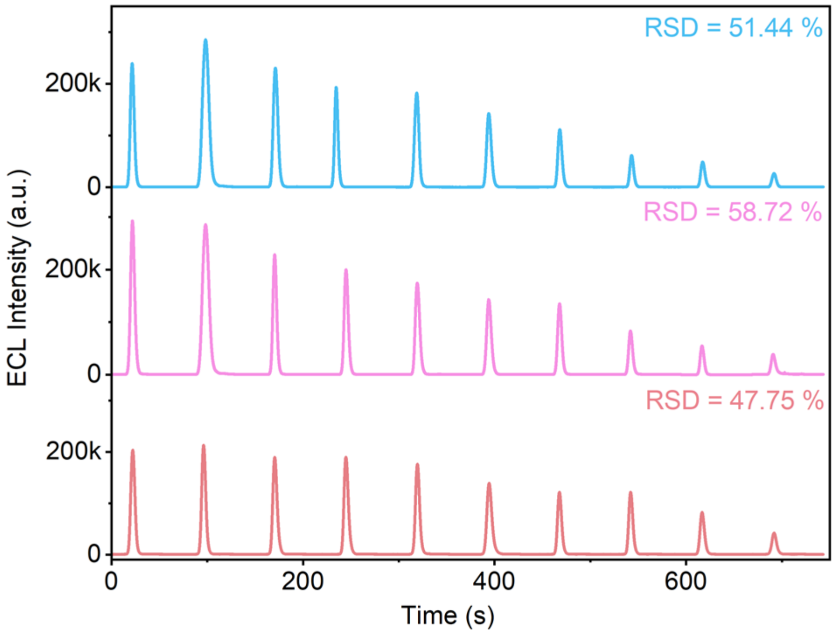

Figure S4. The ECL intensity of Zn^{2+}-AuNCs under continuous cyclic potential scan for 10 cycles.

(8) Cyclic voltammogram of Zn^{2+}-DPA and AuNCs

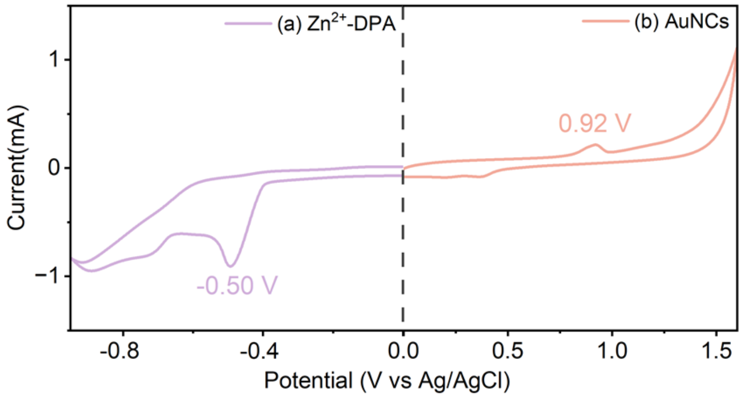

Figure S5. CV profiles of 1.6 mg/mL (a) Zn^{2+}-DPA and (b) AuNCs in 0.1 M KCl solution. Scan rate: 50 mV/s.

(9) PL excitation and emission spectra of AuNCs

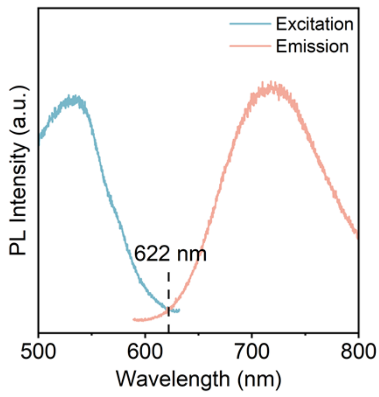

Figure S6. PL excitation and emission spectra (λ_{ex} = 560 nm) of AuNCs in water.

(10) Investigation of the band gap (E_g) and energy level

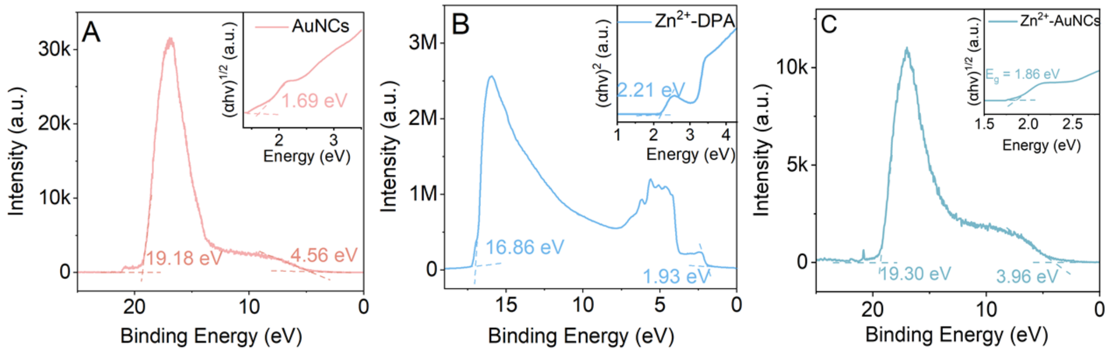

Figure S7. UPS spectra of (A) AuNCs, (B) Zn^{2+}-DPA and (C) Zn^{2+}-AuNCs. Insets of: Taut plots of (A) AuNCs, (B) Zn^{2+}-DPA and (C) Zn^{2+}-AuNCs.

(11) Mott-Schottky curve of Zn^{2+}-AuNCs

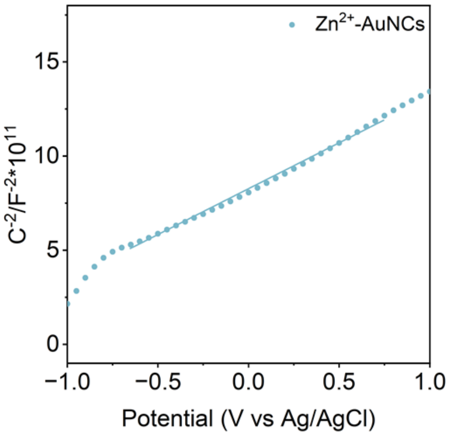

Figure S8. Mott-Schottky curve of Zn^{2+}-AuNCs. Mott-Schottky curve of Zn^{2+}-AuNCs measured in a three-electrode system, with ITO glass coated with Zn^{2+}-AuNCs as the working electrode, Ag/AgCl as the reference electrode, and platinum wire as the counter electrode. The measurement was conducted at an alternating current amplitude of 5 mV and frequencies ranging from 1000 to 3000 Hz in an electrolyte solution of 0.5 M sodium sulfide. The potential scanning range was set from -1.0 V to 1.0 V.

(12) EPR spectrum of Zn^{2+}-AuNCs

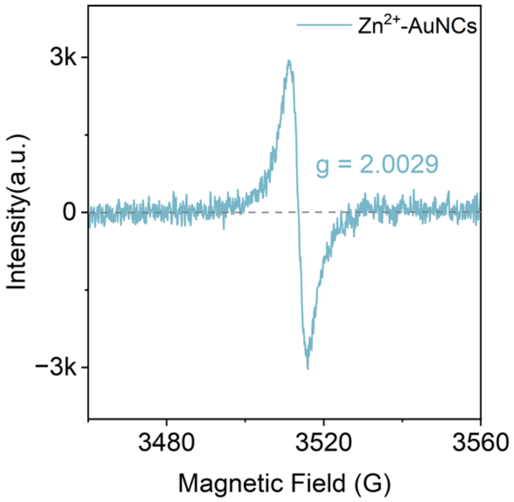

Figure S9. EPR spectrum of Zn^{2+}-AuNCs.

(13) Calculations of energy levels

The energy band (E_g) was estimated using the following formulas:

$$(\alpha h\nu)^{1/n} = k\,(h\nu - E_g) \qquad \text{(eq. 1)}$$

$$E_g = 1240 / \lambda_{onset} \qquad \text{(eq. 2)}$$

Where α, h, ν, k, and E_g were absorption coefficient, Planck's constant, light frequency, a constant and the band gap energy (eV), respectively. The value of the exponent *n* depends on the type of electronic transition in the material: $n = 2$ for direct band gap semiconductors, and $n = 1/2$ for indirect band gap semiconductors.

The energy levels of AuNCs, Zn^{2+}-DPA and Zn^{2+}-AuNCs were calculated according to the UV-photoelectron spectroscopy (UPS) and UV-vis diffuse reflectance spectra.

$$E_{HOMO} = -\,[h\nu + E_{Fermi} - E_{Cutoff}] \qquad \text{(eq. 3)}$$

$$E_{vacuum} = -\,E_{RHE} - 4.44\ \text{eV} \qquad \text{(eq. 4)}$$

$$E_{LUMO} = E_{HOMO} + E_g \qquad \text{(eq. 5)}$$

Where the secondary cutoff energy (E_{Cutoff}) and Fermi energy (E_{Fermi}) of could be calculated using UPS. The value of hν was the incident photon energy (21.22 eV) of He and E_g.
